# Application of a cost-effective DNA extraction protocol for screening transgenic and CRISPR-edited primary goat cells

**DOI:** 10.1371/journal.pone.0239435

**Published:** 2020-09-18

**Authors:** Louhanna Pinheiro Rodrigues Teixeira, Francisco Eder de Moura Lopes, André Saraiva Leão Marcelo Antunes, Matheus Soares Alves, André Marrocos Miranda, Saul Gaudencio Neto, Leonardo Tondello Martins, Ana Cristina de Oliveira Monteiro Moreira, Kaio Cesar Simiano Tavares

**Affiliations:** Experimental Biology Center (NUBEX), University of Fortaleza (UNIFOR), Fortaleza, Ceara, Brazil; University of Bari, ITALY

## Abstract

The genotyping of genetically-modified cells is a crucial step in studies of transgenics and genomic editing with systems such as CRISPR/Cas. The detection of genome editing events can be directly related to the genotyping methodology used, which is influenced by its costs, since many experiments require the analysis of a large number of samples. The aim of this study was to compare the performance of direct lysis methods of genomic DNA (gDNA) extraction for the detection of knockins and knockouts in primary goat cells. Initially, three gDNA extraction protocols (protocol A, heat denaturation/freeze-thaw in water; protocol B, heat denaturation/proteinase K; and protocol C, CellsDirect Kit) were tested using different quantities (1,000, 5,000 and 10,000 cells) and types of goat primary cells (fibroblasts and goat mammary epithelial cells—GMECs) for subsequent validation by PCR amplification of small (*GAPDH*) and large amplicons (hLF transgene). All protocols were successful in the detection of the small amplicon; however, in GMECs, only protocol B resulted efficient amplification (protocol A—0%, protocol B—93%, protocol C—13.33%, P <0.05). In a proof-of-principle experiment, the *TP53* gene was knocked out in GMECs by CRISPR/Cas9-mediated deletion while constructs containing the anti-VEGF monoclonal antibody (pBC-anti-VEGF) and bacterial L-Asparaginase (pBC-ASNase) transgenes were knocked-in separately in fibroblasts. Detection of successful editing was performed using protocol B and PCR. The integration rates of the pBC-ASNase and pBC-anti-VEGF transgenes were 93.6% and 72%, respectively, as per PCR. The efficiency of biallelic editing in GMECs using CRISPR/Cas9 for the *TP53* deletion was 5.4%. Our results suggest that protocol B (heat denaturation/proteinase K) can be used as an inexpensive and quick methodology for detecting genetic modifications in different types of primary goat cells, with efficiency rates consistent with values previously described in the literature when using extraction kits or more complex proteinase K formulations.

## Introduction

Studies in the field of genome editing have enjoyed substantial progress with the development of the CRISPR/Cas9 technology through the quick generation of animal models, stable cell lines and the production of recombinant proteins in cells or transgenic animals. One of the key steps in the process of genome editing is the screening by genotyping of isolated colonies [1–5]. This step may involve a large number of samples, which can reach up to the hundreds, depending on its ultimate utilization or presence of a selection marker, such as reporter or an antibiotic resistance gene [5–7]. For the genotyping, polymerase chain reaction (PCR) has been the most commonly used technique because of its fast results and high precision. However, the reliability of the results obtained by PCR depends on good quality and integrity of the DNA template, which is in turn directly related to the extraction method used. Extraction of genomic DNA (gDNA) using commercially available kits is the method of choice for many researchers [3, 8, 9]. This choice is justified by the more refined methodology of the kits, which mostly include a DNA purification step through silica columns, magnetic beads or ethanol precipitation. Nevertheless, these kits present a limitation of a minimum cell quantity needed to perform the extraction. Moreover, despite their reliability, these kits may be economically disadvantageous and demand excessive handling time when sample volumes are high. Our colleagues [10] estimated a cost of US$ 4,00 per sample for DNA purification with a commercial kit, with the DNA purification column alone costing around US$1,00. As a more affordable alternative, they standardized a homemade column using reusable plastic supporters and filter paper, which dropped the costs to US$ 0,10 per unit [10].

Sample genotyping using DNA extracted with buffer formulations for cell lysis is faster and its cost may be lower than US$ 2,00 per sample [3, 11–13]. Variations of fast and simple cell lysis protocols, such as freeze/thaw in water, alkaline lysis, proteinase K/SDS and commercial buffers have been well described in literature, especially for diagnostic purposes in human samples [14–17]. However, despite the abundance of information available in the literature and the similar biochemical cell composition of different mammalian species, it is still necessary to evaluate the reliability of different DNA extraction protocols regarding the quality and integrity obtained for different kinds of samples to ensure good downstream processing and the reliability of its results.

In addition to the DNA extraction method, another desirable aspect in genotyping is the ability to amplify large DNA fragments (>1000 bp) from genome-edited samples, especially for knockout and knock-in diagnoses [18–20]. The limitations in obtaining large amplicons usually comprise (i) premature stop in the amplification of new strands as a result of sample depuration or erroneous base incorporation in the 3’ extremity and (ii) breaks in the template DNA during the extraction or nuclease degradation [21, 22]. The first limitation can be prevented during the PCR by using a higher pH, glycerol or DMSO addition, reduction of denaturation time, increment of the extension step time and/or use of high-fidelity DNA polymerases [21–23]. The second limitation should be mitigated by choosing and standardizing the ideal lysis method for each sample and downstream processing [24, 25].

In this work, we aimed to validate a quick and cost-effective PCR-based DNA genotyping protocol obtained from cell colonies through the direct lysis of low cell number (less than 10,000). Three different cell lysis techniques were tested for DNA recovery from a low number of goat fibroblast cells and from goat mammary epithelial cells (GMECs). To validate our findings, the best protocol was applied in the genotyping of insertions and deletions in edited fibroblasts and GMECs.

## Materials and methods

### Cell culture

Primary cultures of fibroblasts and epithelial cells from goat mammary glands were used in this study. GMECs were isolated from goat milk as previously described [26]. After the hygiene of the goat’s teats with 1% iodine solution, approximately 200 mL of milk were collected in a sterile container and transported to the laboratory in a cool box with recyclable ice. The milk was diluted 1:2 in DMEM and centrifuged at 300 x g for 15 minutes. The supernatant was discarded and the pellet was resuspended in phosphate buffered saline (PBS) without Ca2+ and Mg2+, followed by centrifugation at 300 x g for 10 min. This washing step was repeated twice. Next, the pellet was resuspended and cultivated in DMEM/F12 (Gibco) medium supplemented with 10% fetal bovine serum (FBS; Gibco), 1% penicillin–streptomycin (Gibco) and 10 ng/mL epidermal growth factor (EGF; Sigma) and incubated at 37.5°C and 5% CO_2_. Fibroblasts were isolated from an ear biopsy [27] of non-transgenic or human lactoferrin (hLF) transgenic goats [28]. Each biopsy was cut into approximately 5 mm pieces and left to rest in six well plates containing DMEM medium (Gibco) supplemented with 10% FBS (Gibco) and 1% penicillin–streptomycin (Gibco) under incubation at 38.5°C and 5% CO_2_ until it was possible to see the presence of explant cells in contact with the plate. Next, the explant was removed and the cells were maintained in culture under the conditions mentioned above. The procedures were approved by the Ethical Commission for Animal Use of University of Fortaleza under protocol number 9572130917.

### Production of transgenic goat fibroblasts

Goat fibroblasts were transfected with the commercial vector pBC-1 Milk Expression Vector (21.6 Kb, cat. no. K270-01, Thermo Fisher Scientific), which promotes random genome integration. The vector was modified by the insertion of a neomycin resistance cassette (1.4 Kb) at the *Not*I site, the 1.03 Kb *Escherichia coli* L-Asparaginase coding sequence (pBC-ASNase, GenBank Gene ID: 947454) and a 1.5 Kb anti-VEGF monoclonal antibody (pBC-anti-VEGF, kindly provided by Dr Jorge Roberto Toledo, from Universidad de Concepción, Chile). A total of 5 x 10^5^ cells were electroporated separately for each DNA construct with 5 μg of the linear vector by applying 1 pulse of 1500 mV for 20 ms using the Neon Transfection System (Thermo Fisher Scientific). Transgenic cells were selected with 600 μg/mL of G418 for 11 to 14 days. At the end of the process, approximately five thousand cells were collected from each colony for DNA extraction and transgene detection by PCR.

### *TP53* deletion in GMEC using CRISPR/Cas9

The GMEC genome was edited with the purpose of promoting a 1229 bp deletion in the *TP53* gene using a pair of CRISPR/Cas9 constructs. Guide RNAs (gRNAs) were designed and submitted to off-target analysis using the Cas-OFFinder software (http://www.rgenome.net/cas-offinder/). Complementary sense and anti-sense oligonucleotides for gRNAs in exons 4 and 7 were commercially synthesized, annealed and cloned in the *Bbs*l site of the pX458 plasmid (Addgene #48138). The pCas9-gRNA4 and pCas9-gRNA7 vectors were co-transfected using Lipofectamine 2000 (Thermo Fisher Scientific) in 2 x 10^5^ GMECs at passage 4 (P4), following the manufacturer’s instructions. Two days after the transfection, cells were passaged in 100-mm plates at a density of 2.5 x 10^4^ cells/plate for clonal isolation of genome-edited cells.

### Experiment 1: Comparison of three DNA extraction methods for the amplification of small and large amplicons

Fibroblasts and GMECs were washed in PBS and separated in groups of 1,000, 5,000 and 10,000 cells (5 technical replicates per group). PBS was removed by centrifugation and pellets were processed according to the recommended protocol for each DNA extraction method.

#### Protocol A: Heat denaturation/freeze-thaw in water

Cells were resuspended in 10 μL of ultrapure DNase/RNase-Free water and heated at 95°C for 10 minutes in a thermal cycler. After cooling on ice, samples were submitted to three cycles of freeze/thaw at room temperature [29]. A total of 2 μL lysate were used in the PCR.

#### Protocol B: Heat denaturation/proteinase K

The method previously described [29] was used with modifications. Each pellet was resuspended in 150 μL of lysis buffer (Tris-HCL 5 mM, pH 8,8). Samples were heated at 95°C for 10 minutes, cooled and incubated at 56°C with 30 μg of proteinase K (Ambion) for 30 minutes, followed by inactivation at 95°C for 10 minutes. Then, samples were centrifuged at 16,000 x g for 1 minute and the supernatant was transferred to another tube. 2 μL were used in the PCR reaction.

#### Protocol C: CellsDirect Resuspension & Lysis Buffers kit

The DNA was extracted using the CellsDirect Resuspension & Lysis Buffers kit (Thermo Fisher Scientific) according to the manufacturer’s instructions. Briefly, cells were resuspended in a mix of Resuspension Buffer and Lysis Enhancer at a proportion of 10:1, respectively, and incubated at 75°C for 10 minutes. A total of 2 μL were used for the PCR.

#### PCR amplification of small and large amplicons

The *GAPDH* (Glyceraldehyde 3-phosphate dehydrogenase) gene was the target for the amplification of a 150 bp product in both cell types. The amplification of a larger amplicon was evaluated in fibroblasts using the pBC-F and pBC-R primers that anneal to the pBC-1 vector and flank the hLF transgene, generating a 1,500 bp amplicon. PCR was performed using *Platinum Taq DNA Polymerase* (Thermo Fisher Scientific) according to the manufacturer’s instructions. The reaction was incubated at 95°C for 5 min, 35 cycles each with 95°C for 30 s, 60° C for 30 s, and 72°C for 1 min (pBC primers) or 30 s (*GAPDH* primers), followed by a final elongation step at 72°C for 5 min. In GMECs, primers located at the *TP53* gene were used for amplification of a 1746 bp product. The PCR efficiency was also evaluated after a cycle of freezing and thawing with storage at -20°C. All primers used are listed in Table 1.

**Table 1:** Target sequence of the gRNAs, primer sequences and amplicon sizes.

Primer/Gene	Sequence 5´ - 3´	Amplicon size (bp)
gRNA 4	GCCTCCTGCCCAAGCTGCCC	-
gRNA 7	CCTGCATGGGGGGCATGAAC	-
*GAPDH*	F- GATTGTCAGCAATGCCTCCT	150
	R- AAGCAGGGATGATGTTCTGG	
pBC	F- GATTGACAAGTAATACGCTGTTTCCTC	1500
	R- CATCAGAAGTTAAACAGCACAGTTAG	
*TP53*	F- TAGAGGCCTGGGAGAAACAA	1746
	R- ATTGAGACGATCCCAGCAAG	
ASNase	R- ACGTTGGCGATATCCTTCAG	300
	pBC-F	
Anti-VEGF	F- TACACTCTCCCTCCTAGTCG	350
	pBC-R	

### Experiment 2: Genotyping of transgenic and CRISPR-edited primary goat cells

Results from experiment 1 indicated that gDNA extraction with protocol B was the most efficient. Thus, this protocol was chosen for a proof-of-principle genotyping of transgenic fibroblasts and *TP53* knockout GMECs. The PCR for knockin diagnoses in fibroblasts was performed using primer pairs that anneal to the vector backbone flanking the transgene. The analysis of *TP53* knockout in GMEC was done with primers that bind upstream of the gRNA4 target and downstream of the gRNA7 target. The sequence of gRNAs and of all primers are listed in Table 1. All reactions were carried out in 20 μL reactions using 2 μL of lysate, 2 mM of Mg^2+^, 0.5 mM of dNTP mix, 0.5 μM of each primer and 2 units of Platinum Taq DNA polymerase (Thermo Fisher Scientific). Cycling conditions were set according the manufacturer’s instructions and repeated 35 times. PCR products were analyzed in 1% agarose gel. Fibroblast colonies with transgene insertions and GMECs with allelic disruption were confirmed by direct sequencing of PCR products. DNA sequences were analyzed using the SnapGene software (Free Trial).

### Statistical analyses

Statistical analyses were carried out with Fisher’s exact test using the Social Science Statistics software (https://www.socscistatistics.com/tests/fisher/default2.aspx). The analyses were performed on the results of amplification efficiency of small and large DNA sizes between different lysis methods and different types of goat cells (fibroblasts and GMECs).

## Results

### Evaluation of direct lysis methods for goat fibroblasts and GMEC

**Fig 1:**
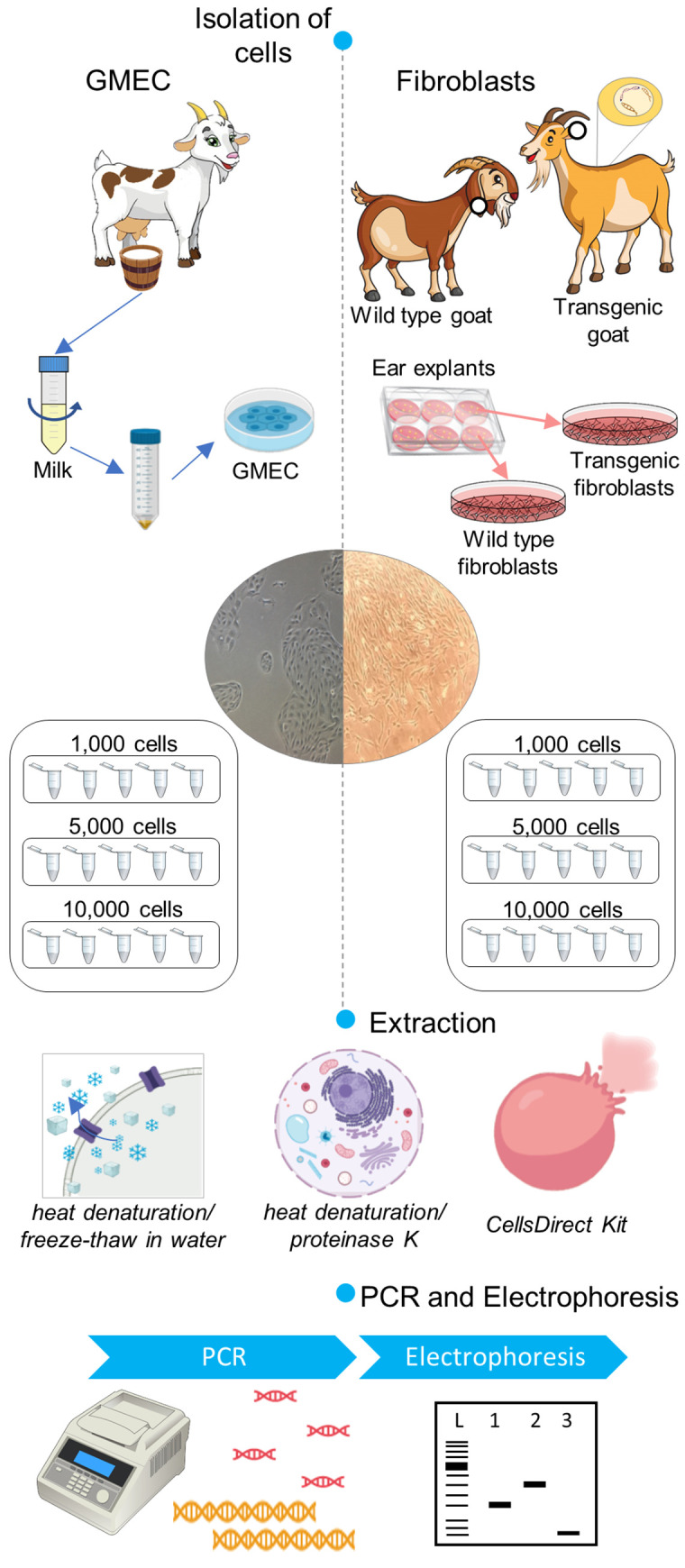
Workflow of the standardization step of the lysis methods for goat cells. First, GMECs, wild type and transgenic fibroblasts were isolated from goat milk, ear explants of wild type goat and ear explants of transgenic goat (for the human lactoferrin gene), respectively. Primary culture of GMEC and fibroblasts were divided into groups of 1,000, 5,000 and 10,000 cells (n = 5) and submitted to DNA extraction using protocol A, heat denaturation/freeze-thaw in water; protocol B, heat denaturation/proteinase K; and protocol C, CellsDirect Kit. Finally, PCR was carried out using primers for a small amplicon (*GAPDH*) and a large amplicon (*TP53* and *hLF*, for GMEC and fibroblasts, respectively).

**Fig 2:**
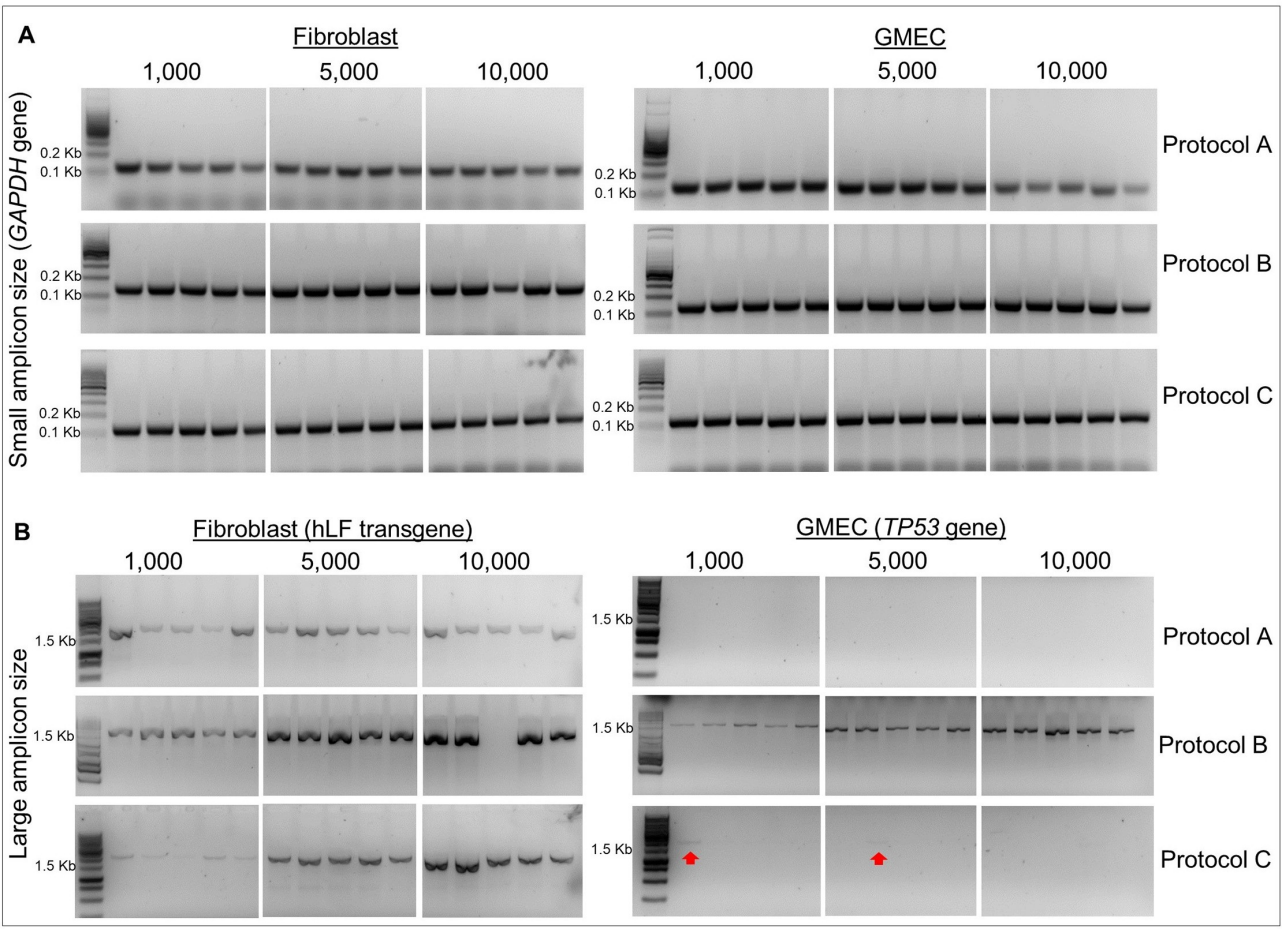
PCR analysis of experiment 1 (comparison between lysis protocols A, B and C). (A) PCR of the *GAPDH* gene for the amplification of small fragments (150 bp) in fibroblasts and GMEC. (B) PCR for the amplification of large fragments. The transgene *hLF* (1,500 bp) was amplified in fibroblasts. The target in GMEC was a portion of the *TP53* gene (1746 bp).

The DNA extraction efficiency between three cell lysis methods using three different cell numbers (1,000, 5,000 and 10,000 cells) of two types of primary cells (GMECs and goat fibroblasts) was measured by PCR of small and large amplicons (the workflow is illustrated in Fig 1). All methods (protocol A, heat denaturation/freeze-thaw in water; protocol B, heat denaturation/proteinase K; and protocol C, CellsDirect Kit) yielded DNA with good quality for product amplification of around 150 bp (Fig 2A and Table 2) in both cell types. DNA integrity analysis for amplification of large fragments was performed by PCR of the *hLF* transgene present in transgenic fibroblasts and of the *TP53* gene in GMECs. The amplification of large fragments, 1,500 bp for *hLF* and 1,746 bp for *TP53*, is shown in Fig 2B and Table 2. For fibroblasts, the amplification of large amplicons was observed in all lysis methods and for all cell quantities evaluated, without any statistical difference between groups (P>0,05). However, for GMECs, protocol B was the only one that showed high amplification efficiency for a large amplicon size (93,3%), which is significantly different from the other groups for all cell numbers analyzed (P<0,05). All PCRs were repeated after a freezing and thawing cycle, when the cells were stored at -20°C and the results were similar (S1 Fig and S1 Table). Thus, lysis with proteinase K (protocol B) was the chosen method for DNA extraction in the following genotyping step.

**Table 2:** Number of amplified samples in PCR for large and small amplicons using protocol A, heat denaturation/freeze-thaw in water; protocol B, heat denaturation/proteinase K; and protocol C, CellsDirect Kit.

DNA extraction	Cell type	Large amplicon size positive PCR			Small amplicon size positive PCR		
		1,000 cells	5,000 cells	10,000 cells	1,000 Cells	5,000 cells	10,000 cells
Protocol A	Fibroblast	5/5^a^^,^^A^	5/5^a^^,^^A^	5/5^a^^,^^A^	5/5^a^^,^^A^	5/5^a^^,^^A^	5/5^a^^,^^A^
	GMEC	0/5^b^^,^^B^	0/5^b^^,^^B^	0/5^b^^,^^B^	5/5^a^^,^^A^	5/5^a^^,^^A^	5/5^a^^,^^A^
Protocol B	Fibroblast	5/5^a^^,^^A^	5/5^a^^,^^A^	4/5^a^^,^^A^	5/5^a^^,^^A^	5/5^a^^,^^A^	5/5^a^^,^^A^
	GMEC	5/5^a^^,^^A^	5/5^a^^,^^A^	5/5^a^^,^^A^	5/5^a^^,^^A^	5/5^a^^,^^A^	5/5^a^^,^^A^
Protocol C	Fibroblast	5/5^a^^,^^A^	5/5^a^^,^^A^	5/5^a^^,^^A^	5/5^a^^,^^A^	5/5^a^^,^^A^	5/5^a^^,^^A^
	GMEC	1/5^b^^,^^B^	1/5^b^^,^^B^	0/5^b^^,^^B^	5/5^a^^,^^A^	5/5^a^^,^^A^	5/5^a^^,^^A^

^a,b:^ Numbers with distinct superscripts in the column differ, with P<0.05.

^A,B:^ Numbers with distinct superscripts in the row differ, with P<0.05.

### Production and diagnosis of transgenic goat fibroblasts and CRISPR-edited GMECs

**Fig 3:**
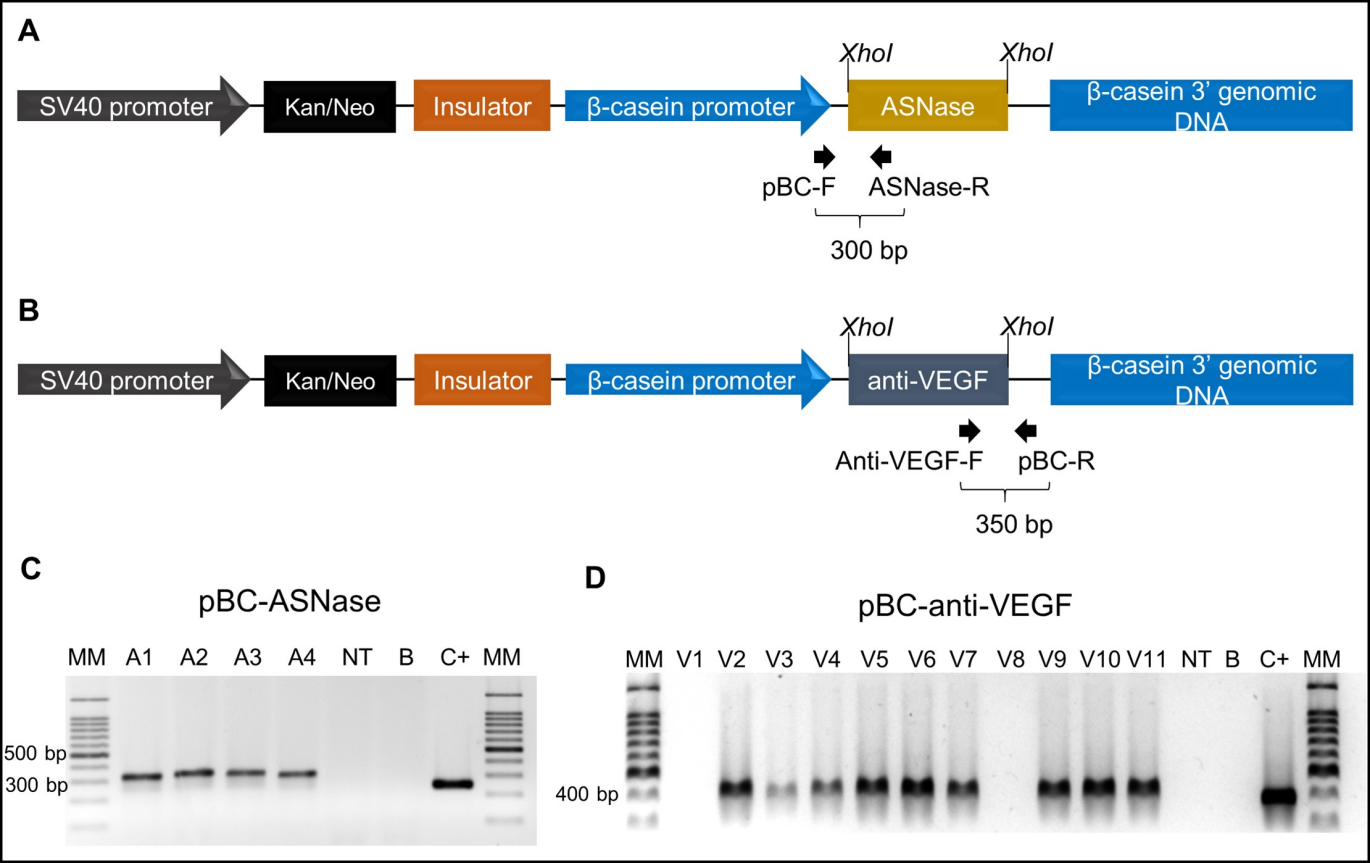
Construction of the donor vectors for knockin and PCR genotyping of transgenic fibroblasts colonies. (A) Representation of the pBC-ASNase and (B) pBC-anti-VEGF constructs. In both schemes it is possible to observe the kanamycin and geneticin (kan^R^) resistance gene and the beta-casein promoter. (C) and (D) PCR analysis of the insertion of the plasmids pBC-ASNase and pBC-anti-VEGF in fibroblasts. Expected amplicon of 300 bp and 350 bp, respectively. MM: 100 bp ladder, A1 to A4: Fibroblast colonies transfected with pBC-ASNase; V1 to V11: Fibroblast colonies transfected with pBC-anti-VEGF; NT: DNA from a non-transgenic fibroblast; B: H_2_O ultrapure; C+: positive control (pBC-ASNase and pBC-anti-VEGF plasmids).

The donor vectors pBC-ASNase and pBC-anti-VEGF were constructed and electroporated into primary goat fibroblasts; transgenic cells were selected through the resistance gene that was present in the vector (Fig 3A and 3B). In addition, the vector carries the chicken beta-globin insulator sequence (depicted in Fig 3), to avoid transgene position effect, and lacks homology arms for HDR. Using protocol B for DNA extraction and PCR yielding amplicons of 300 bp and 350 bp for PBC-ASNase and PBC-anti-VEGF, respectively, a total of 93.6% of the colonies for pBC-ASNase, (103/110) and 82% of the colonies isolated for pBC-anti-VEGF (44/61) were diagnosed as transgenic.

**Fig 4:**
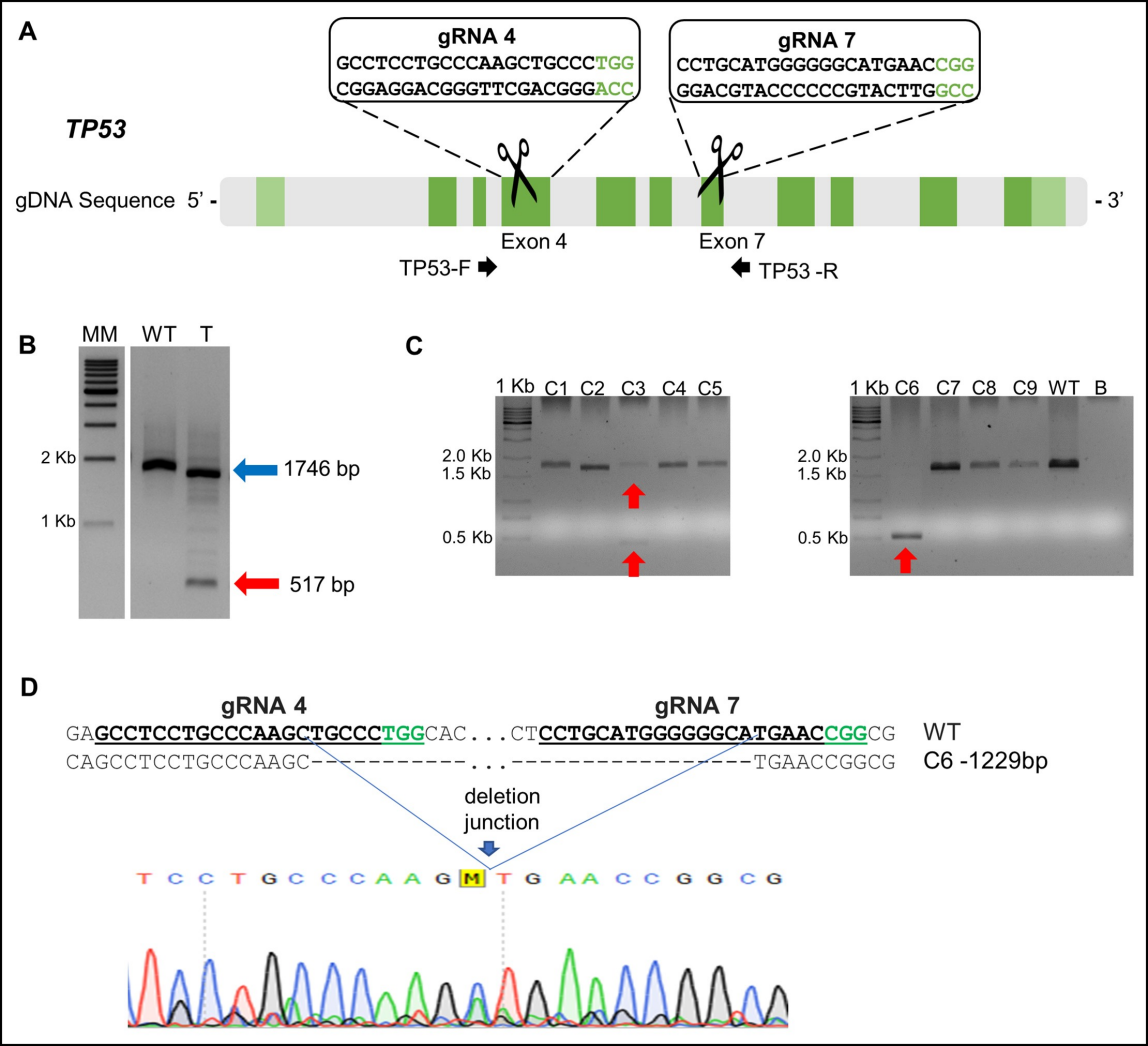
Target sequences of CRISPR/Cas9 for *TP53* deletion and genotyping of isolated colonies. (A) Sequences of the two gRNAs + PAM with target at exons 4 and 7 of the goat *TP53* gene. (B) PCR for the analysis of fragment deletion for CRISPR/Cas9 pair in GMEC pool. MM: 1 Kb ladder, WT: GMEC wild type and T: co-transfected GMEC cell pool. The blue arrow indicates the 1,746 bp amplicon referring to the non-edited DNA sequence. The red arrow corresponds to a 517 bp amplicon from an allele with fragment deletion that occurred by cleavage of both CRISPRs. (C) Genotyping of GMEC colonies by PCR. 1,746 bp band indicates the allele without genic editing and the 517 bp band corresponds to the allele with deletion caused by both CRISPR constructs. Colony C3 showed a monoallelic deletion and C6 showed a biallelic deletion of *TP53*. 1 KB: 1 Kb ladder; C1 to C9: GMEC colonies co-transfected with pCas9-gRNA4 and pCas9-gRNA7; WT: DNA from non-transfected GMECs, wild type; B: H_2_O ultrapure. (D) Sequence analysis of the target *TP53* genomic loci wild type (WT) and C6 colony showing the expected 1,231 bp deletion.

The two CRISPR/Cas9 systems (pCas9-gRNA4 and pCas9-gRNA7) targeting the goat *TP53* gene were co-transfected into GMEC and deletion events were evaluated by PCR with primers flanking the deleted region with DNA being extracted using protocol B (Fig 4A). A reduction in the amplicon size, from 1,746 bp to 517 bp (1,229 bp deleted) was observed, indicating that both CRISPR/Cas9 systems cleaved in the same allele of the *TP53* gene (Fig 4B). After GMEC clonal isolation by limiting dilution, a total of 2 out of 37 colonies (5.4%) were diagnosed with TP53 deletion by large fragment exclusion. Of note, small indel mutations are not the focus of our work, thus we did not assess the rate of this kind of edition.

Of the two positive GMEC colonies, one showed a biallelic edition (*TP53*^−/−^) and the other a monoallelic edition (*TP53*^+/−^) (Fig 4C). This result was confirmed by DNA sequencing (Fig 4D).

## Discussion

In the genomic engineering context, different technologies are applied for the generation of knockins and knockout of genes and regulatory sequences in the DNA. The genotyping of numerous samples using a few cells is a key step in the screening process and very often it can be costly, mainly when commercial kits are used for genomic DNA purification. In this study, we investigated three methods used for the lysis of a small number of cells. The buffers for two of those methods were ‘homemade’ and a commercial kit was used as a third method. The three DNA purification methods resulted in good-quality DNA from goat fibroblasts, allowing the amplification of up to 1500 bp. However, when we analyze only large amplicons, the intensity of the bands seen in the agarose gel for PCR from protocol A extraction was lower when compared to protocol B. The same was observed in samples obtained from 1,000 cells extracted with protocol C. For goat mammary gland cells, the successful amplification of 1,746 bp was observed only for protocol B lysis (heat denaturation/proteinase k). Interestingly, all lysis methods amplified small products (150 bp) with strong intensity when observed in agarose gel, regardless of the cell quantity evaluated in both cell types.

Our hypothesis is that the less efficient downstream processing of DNA observed in protocol A and C in GMECs was probably due to (i) the presence of PCR inhibitors—that act directly or indirectly on in the DNA polymerase or nucleic acid sequestering by proteins and nucleases which bind to DNA [24]—or (ii) the presence of active nucleases in the samples that caused the fragmentation of large extracted DNA strands. The first hypothesis seems to be less likely, due the observation of a lack of influence of the lysis methods resulting from the small amplicons’ amplification. Furthermore, the presence of plasmin and calcium ions, PCR inhibitors present in milk [24] should not occur in GMEC cultivated *in vitro*, since this protease reaches the mammary tissue from the blood [30] and the calcium ions would have been removed during the previous cell washes in PBS. Nevertheless, we cannot rule out the possibility of the presence of inhibitor molecules stored or produced by the cell itself. The second hypothesis is supported by the tissue’s own characteristics, such as alteration in the number of cells, size, structure, composition and activity when referring to the different phases of gestation, lactation and involution. The regulation of the transition between those phases involves the alteration of the nuclease content to repair or degrade the DNA [31]. Furthermore, our colleagues [32] report the presence of tissue-specific DNases and extracellular DNAses in epidermal cells, which are involved in the defense against infectious agents [32]. As GMECs are related to the epidermal cells, considering their epithelial origin, we consider the possibility of the presence of a higher nuclease content in those cells when compared to fibroblasts. We suspect that the temperature and denaturation time for heating in protocol A and C were not enough to completely eliminate nuclease activity in GMECs without the presence of proteinase K. Thus, our results suggest that special attention should be given to the DNA extraction method chosen depending on the cell type used.

The security offered by commercial DNA extraction kits as to molecule integrity and purity is sometimes surpassed by the high final costs, resulting from the number of samples to be analyzed, which in some cases or applications are considerably high. In our work, protocol B had a mean cost of about US$ 1.2 per sample, which represents a cost reduction of 70% over the one presented by our colleagues [10]. In addition, this protocol was performed in less than 50 min, while commercial kits usually take over 1 hour. In search of these advantages, some studies in the genomic editing field with a large number of sample analyses are shifting towards ‘homemade’ methods for DNA extraction. For instance, experiments in goats [5] and cattle [11] were conducted using a variation of lysis protocols with proteinase K for the screening of 121 and 47 colonies, respectively, by PCR or PCR-RFLP. Editing efficiency in both studies were 100% and 31,5%. Although these efficiencies are closely related to factors such the gene editing tools used, DNA repair pathways and transfection rate, the genotyping method with direct lysis using proteinase K showed to be functional for this kind of diagnosis, as observed in our results. Even so, differently from the buffer used here (5 mM of Tris-HCI pH 8,8 supplemented with 30 μg of proteinase K), the solutions used by those groups comprised a larger number of constituents, such SDS, EDTA and NaCl, as well as a longer incubation time. The simple lysis buffer composition consisting only of Tris-HCl and proteinase K has as an advantage the absence of compounds (SDS, DTT, EDTA, and NaCl) that, at high concentrations, may inhibit the PCR [12, 24].

After the standardization and comparison of three different cell lysis protocols, we applied protocol B for the genotyping of three genomic modifications in goat cells: (i) knockin of the pBC-ASNase transgene in fibroblasts, (ii) knockin of pBC-anti-VEGF in fibroblasts and (iii) knockout of the *TP53* gene in mammary gland cells. In the knockin experiments, 93.6% of the colonies transfected with pBC-ASNase and 73% of the colonies transfected with pBC-anti-VEGF were diagnosed with transgene integration. In a study for the production of a bitransgenic goat, an integration efficiency of 86.5% was observed for the human transgene CuZn-SOD and 50% for the human transgene EC-SOD; the simultaneous integration efficiency of the two transgenes was 43.2% [6]. Feng et al. reached a 100% integration efficiency for the human transgene a-lactalbumin in goat fibroblasts after selecting 121 colonies with two markers, resistance to G418 and GFP expression [5]. These studies, similar to ours, showed high knockin levels; additionally, they also had in common the selection of colonies by G418 resistance and genotyping of a few cells. Although the DNA extraction method used by them was not clear, the authors performed cell lysis with a solution containing proteinase K and SDS only [5, 6].

Aiming to produce CRISPR/Cas9 systems that allow for the *TP53* gene knockout in goat mammary gland cells, we had difficulties related to the validation of the target cleavage efficiency of two CRISPR/Cas9 systems using the T7E1 assay. In fact, there are characteristic limitations for the mismatch recognition techniques (Surveyor and T7E1) as allelic variants and the incapacity to differentiate alleles with identical mutations (false wild type) [33, 34]. Alternatives for those diagnoses are techniques such as fluorescent PCR [35], PCR with overlapping primers in the cleavage site [36], RGEN-mediated RFLP analysis [34], high resolution melting analysis (HRM) [37], Sanger sequencing [38] and deep sequencing [39]. However, we used a strategy that we consider to be simpler: PCR analysis using primers flanking the deleted fragment located between two both cleavage targets. For this purpose, we co-transfected a GMEC group with plasmids of the two CRISPR/Cas9 systems carrying gRNAs for exons 4 and 7 and validated the target cleavage efficiency of both systems after repair through non-homologous end joining (NHEJ) (Fig 3D). The use of this strategy has advantages, as the molecular design and cloning of an additional CRISPR is fast and low cost and at the end of the process the researcher will have two CRISPR options to use in additional experiments, i.e. transgene knockin. The disadvantage of this method lies on the possibility of having a diagnosis only when there is cleavage efficiency in more than one target. Nevertheless, this strategy is extremely simple and reliable regarding the alteration of the reading frame.

## Conclusions

This study validated a direct lysis method for DNA extraction and subsequent amplification of genomic segments by PCR from a low number of cells in two types of goat primary cells (fibroblasts and GMECs). The efficiency of the protocols varied with cell type and amplicon size. Protocol B (heat denaturation/proteinase K) was the only one that showed satisfactory results in all conditions. Finally, protocol B was successfully used for the knockin diagnosis of two transgenes in fibroblasts and the deletion of the segment located between exons 4 and 7 and the *TP53* gene in GMECs.

## Supporting information

S1 Fig PCR analysis of experiment 1 (comparison between lysis protocol A, B and C) after a freezing and thawing cycle.
(TIFF)

S1 Table Number of amplified samples in PCR for large and small amplicons after a freezing and thawing cycle. (DOCX)
