# Supplementary figures and images for: Application of a cost-effective DNA extraction protocol for screening transgenic and CRISPR-edited primary goat cells
Source: PLoS One. 2020 Sep 18;15(9):e0239435. doi: 10.1371/journal.pone.0239435 (PMC7500585; doi:10.1371/journal.pone.0239435)

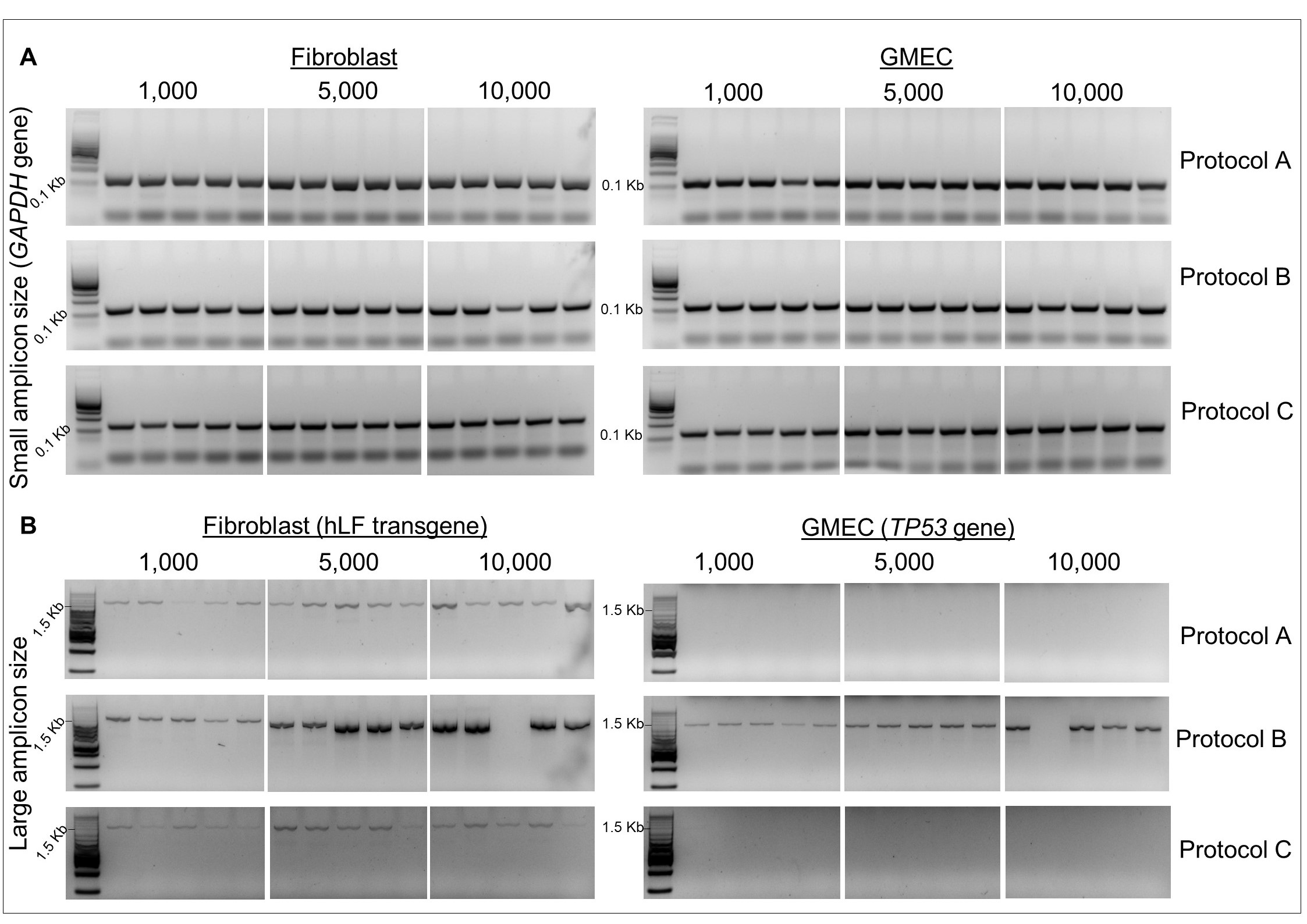

Supplement: S1 Fig — PCR analysis of experiment 1 (comparison between lysis protocol A, B and C) after a freezing and thawing cycle. (TIFF) [file pone.0239435.s001.tiff]
