# Supplementary material for: Application of a cost-effective DNA extraction protocol for screening transgenic and CRISPR-edited primary goat cells
Source: PLoS One. 2020 Sep 18;15(9):e0239435. doi: 10.1371/journal.pone.0239435 (PMC7500585; doi:10.1371/journal.pone.0239435)
Supplement: S1 Table — (DOCX) [file pone.0239435.s002.docx]

**S1 Table.** **Number of amplified samples in PCR for large and small amplicons after a freezing and thawing cycle.**

| **DNA extraction** | **Cell type** | **Large amplicon size positive PCR** | | | **Small amplicon size positive PCR** | | |
| --- | --- | --- | --- | --- | --- | --- | --- |
|  |  | **1,000 cells** | **5,000 cells** | **10,000 cells** | **1,000 Cells** | **5,000 cells** | **10,000 cells** |
| Protocol A | Fibroblast | 5/5^a,A^ | 5/5^a,A^ | 5/5^a,A^ | 5/5^a,A^ | 5/5^a,A^ | 5/5^a,A^ |
|  | GMEC | 0/5^b,B^ | 0/5^b,B^ | 0/5^b,B^ | 5/5^a,A^ | 5/5^a,A^ | 5/5^a,A^ |
| Protocol B | Fibroblast | 5/5^a,A^ | 5/5^a,A^ | 4/5^a,A^ | 5/5^a,A^ | 5/5^a,A^ | 5/5^a,A^ |
|  | GMEC | 5/5^a,A^ | 5/5^a,A^ | 4/5^a,A*^ | 5/5^a,A^ | 5/5^a,A^ | 5/5^a,A^ |
| Protocol C | Fibroblast | 5/5^a,A^ | 5/5^a,A^ | 5/5^a,A^ | 5/5^a,A^ | 5/5^a,A^ | 5/5^a,A^ |
|  | GMEC | 0/5^b,B*^ | 0/5^b,B*^ | 0/5^b,B^ | 5/5^a,A^ | 5/5^a,A^ | 5/5^a,A^ |

Protocol A, heat denaturation/freeze-thaw in water; protocol B, heat denaturation/proteinase K; and protocol C, CellsDirect Kit.

* Observed changes on PCR between the analysis before and after the freezing process are detached in yellow.

^a,b:^ Numbers with distinct superscripts in the column differ, with P<0.05.

^A,B:^ Numbers with distinct superscripts in the row differ, with P<0.05.
